# Ahi1 regulates the nuclear translocation of glucocorticoid receptor to modulate stress response

**DOI:** 10.1038/s41398-021-01305-x

**Published:** 2021-03-29

**Authors:** Bin Wang, Ning Xin, Xuanchen Qian, Lijing Zhai, Zhigang Miao, Yong Yang, Shihua Li, Miao Sun, Xingshun Xu, Xiao-Jiang Li

**Affiliations:** 1 Institute for Fetology, The First Affiliated Hospital of Soochow University, 215006 Suzhou, China; 2 Institute of Neuroscience, Soochow University, 215123 Suzhou, China; 3 Department of Neurology, The Affiliated Hospital of Xuzhou Medical University, 221000 Xuzhou, Jiangsu China; 4 Department of Psychiatry, The Affiliated Guangji Hospital of Soochow University, 215008 Suzhou, China; 5 Guangdong Key Laboratory of non-human primate models, Guangdong-Hongkong-Macau Institute of CNS Regeneration, Jinan University, 510632 Guangzhou, China; 6 Department of Neurology, The First Affiliated Hospital of Soochow University, 215006 Suzhou, China; 7 Jiangsu Key Laboratory of Neuropsychiatric Diseases, Soochow University, 215123 Suzhou, Jiangsu China

**Keywords:** Molecular neuroscience, Pathogenesis

## Abstract

Stress activates the nuclear translocation of glucocorticoid receptors (GR) to trigger gene expression. Abnormal GR levels can alter the stress responses in animals and therapeutic effects of antidepressants. Here, we reported that stress-mediated nuclear translocation of GR reduced Ahi1 in the stressed cells and mouse brains. Ahi1 interacts with GR to stabilize each other in the cytoplasm. Importantly, Ahi1 deficiency promotes the degradation of GR in the cytoplasm and reduced the nuclear translocation of GR in response to stress. Genetic depletion of Ahi1 in mice caused hyposensitivity to antidepressants under the stress condition. These findings suggest that AHI1 is an important regulator of GR level and may serve as a therapeutic target for stress-related disorders.

## Introduction

Stress activates the hypothalamic–pituitary–adrenal (HPA) axis to release glucocorticoid hormones from the adrenal cortex, which cross the blood–brain barrier and target mineralocorticoid receptors and glucocorticoid receptors (GR) in the brain. Upon ligand binding, GR is translocated from the cytoplasm to the nuclei to trigger stress response signaling pathways^1–3^. Thus, impaired GR signaling is considered an important contributor to stress-related disorders, such as major depressive disorders (MDD)^4,5^, and targeting impaired GR functions is a therapeutic mechanism of different antidepressants^6,7^. However, how the GR signaling pathway is involved in stress-related disorders and their treatments remains to be fully investigated.

The Abelson helper integration site 1 (*AHI1*) gene plays a pivotal role in brain development, and the mutations of *AHI1* cause Joubert syndrome, a neurodevelopmental disorder^8^. Recent studies have indicated that *AHI1* is a high susceptibility gene for schizophrenia^9–12^ and autism^13^, two common neuropsychological disorders with depressive symptoms, and is also linked to human populations with depressive disorders^14^. Consistently, Ahi1 deficiency in mice is associated with depressive-like behaviors^11,15^ and a stress-resilient phenotype^16,17^. However, we still lack direct knowledge of the mechanisms for an association between Ahi1 and depressive-like behaviors.

In the current study, we found that Ahi1 is reduced in the brain of the stressed mice with depressive-like behaviors and that this reduction could be reversed by antidepressant treatment. To delineate the mechanism behind these phenomena, our studies show that Ahi1 interacts with GR to regulate its translocation from the cytoplasm to the nuclei, thereby modifying GR-mediated stress response.

## Materials and methods

### Antibodies and reagents

Ahi1 Rabbit Antibody and Hap1 Guinea pigs Antibody were described previously^18^; GR antibody (sc-56851) and His-probe antibody (sc-53073) were obtained from Santa-Cruz Biotechnology Inc. (Santa-Cruz, CA, USA); LSD-1 (#2139), and Histone-3 (#4499) antibodies were from Cell Signaling Technology, Inc., (Danver, MA, USA);ubiquitin antibody was from Abcam, (ab134953,Cambridge, MA, USA), Beta-tubulin antibody, anti-beta-actin antibody, imipramine (IM), mifepristone (RU 38486), fluoxetine, MG132, and dexamethasone acetate (Dex) were from Sigma-Aldrich Company (St. Louis, Missouri, USA); RPMI-1640 and FBS (Gibco Company, MD, USA); ECL chemiluminescence system was from Thermo Company (West Chester, PA, USA); Mouse corticosterone ELISA kit was purchased from Cusabio Biological Engineering Co. LTD (Baltimore, MD, USA); Transcriptor First Strand cDNA Synthesis Kit and 2XSYBR Green PCR Master Mix (Roche, Germany); Lipofectamine 2000 was bought from Invitrogen Corporation (San Diego, CA, USA). All secondary antibodies were purchased from Jackson ImmunoResearch Laboratories (West Grove, PA, USA).

### Plasmid construction

PRK-Ahi1-HA plasmid was generated previously^18,19^. Full-length mouse AHI1 (aa 1–1047) was produced by PCR of mouse cDNA^20^. We also used PCR to generate truncated mouse Ahi1, including N-terminal Ahi1 (nAhi1, aa 1–284), Ahi1 protein without N-terminal region (ΔnAhi1, aa 285–1047), and C-terminal Ahi1 (cAhi1, aa 651–1047). Ahi1 cDNAs were inserted into the pCDNA3.1 Eukaryon vector that links His tag to the expressed Ahi1. These plasmids were transfected into PC12 cells by Lipofectamine 2000 to express Ahi1-His fusion proteins. The construction methods of full-length rat pCDNA3.1-GR-GST plasmid and full-length mouse pET30a-His-GR were the same as that of Ahi1 plasmid.

### Animals

Ahi1 knockout (KO, Ahi1^−/−^) mice were generated as described previously^15,21^, Ahi1^loxp/loxp^ mice were crossed with mice carrying an EIIa promoter-driven Cre transgene, and the resulting heterozygous mice were used to generate homozygous conditional KO mice. About 2-months-old Ahi1 mice were used in the experiments. Male ICR mice (25–30 g) at age of 2 months old were bought from SLAC Company (Shanghai, China). Mice were maintained in a 12-h light/dark cycle in a specific pathogen-free animal housing at Soochow University Experimental Animal Center.

### Mouse stress models

Chronic glucocorticoid exposure has been shown to induce a depressive-like motivational state in rodents that is similar to that produced by a chronic mild stress paradigm^22^. To induce depressive behaviors in mice, 2-month-old male ICR mice (25–30 g) were exposed to subcutaneous injection of Dex (1 mg/kg) or vehicle daily for 21 days.

For the spatial restraint stress model, mice were placed in a 50-ml tube for space restriction at 09:00–11:00 daily; control mice were placed in the cage without the access to water and food at the same time period as descried previously^23^. The mice were randomly divided into control group and stress group. After mice were treated by stress and verified by behavioral tests, the mice that were very sick were excluded from the study. For antidepressant treatment, mice were randomly assigned to four groups: saline (NS) group, Imipramine (IM) group, Stress+NS group, and Stress+IM group. After one week of spatial restraint stress, IM (20 mg/kg) was freshly prepared in normal saline and intraperitoneally injected 30 min before spatial restraint stress daily for 3 weeks. Saline-treated mice received same volume of normal saline.

### Chronic social defeat stress (CSDS)

CSDS was performed according to the previous studies with minor modifications^24,25^. In brief, the mice were subjected to social frustration for 10 consecutive days. In the CSDS experiment, we used two stains of mice (CD1 and ICR) and CD1 strain mice to attack ICR mice. Every day, each mouse was introduced into the home cage of a stranger resident. Resident mice were CD1 retired breeders selected for their attack latencies reliably shorter than 30 s upon three consecutive screening tests. Once the experimental mice were physically defeated in three attacks, both animals (defeated and aggressor) were divided into two parts by a metal mesh, maintaining 24-h sensory contact. Within 10 days, the control mice were placed in the same cage alone, but the other half contained members of the same strain.

### Forced swim test (FST)

Forced swim test was performed as described previously^23^. Each mouse was placed in a glass cylinder (20 cm high, 15 cm in diameter) with water (23–25 °C) about 14 cm in depth. The videos of swim test were taken in a 6-min section. The immobility time was recorded by a trained person with a stopwatch.

### Tail suspension test (TST)

Mice were suspended for 6 min by sticking the tip of the tail to a rod above the desktop about 35 cm. Normal mice show struggle behaviors. The immobility time without struggling was recorded.

### Sucrose preference test (SPT)

Mice were housed individually and trained to drink from two bottles with water for 24 h. In the next day, water in one bottle was replaced with 1% sucrose. In the third day, the positions of two bottles were exchanged. In the fourth day, mice were deprived of water and food for 24 h. In the fifth day, mice were freely accessed to drink and the consumption of water/1% sucrose was recorded by weighing the bottles. The percentage of 1% sucrose consumed in the fifth day was calculated.

### PC12 cell culture and plasmid transfection

PC12 cells were maintained in RPMI-1640 containing 10% fetal bovine serum, 100 UI/ml penicillin sodium, and 100 μg/ml streptomycin sulfate. When PC12 cells reached 50–60% of the confluence, the medium was changed to phenol red-free medium supplemented with 10% charcoal-treated FBS to reduce endogenous steroids. Plasmid (DNA 1 μg/well) was mixed with 50 μl serum-free medium. At the same time, 2 μl Lipofectamine 2000 was mixed with 50 μl serum-free medium. After plasmid DNA and Lipo2000 were mixed and incubated for 20 min at room temperature, 100 μl of the mixture and 900 μl serum-free medium was added to each well of 6-well plates. After the incubation for 6 h, the medium was replaced with regular culture medium. At 48 h after the transfection, PC12 cells were collected for western blotting analysis.

### Western blotting analysis

Mouse brain tissues or PC12 cells were solubilized in lysis buffer. After centrifuging, supernatants were collected and protein concentrations were determined. Protein samples were loaded on a sodium dodecyl sulfate-polyacrylamide gel electrophoresis and then electro-transferred to nitrocellulose membranes. Membrane blots were blocked with 5% dry milk in phosphate-buffer saline/0.1%Tween 20 (PBST) for 2 h. After the incubation with primary antibodies with shaking at 4 °C overnight, membrane blots were washed and incubated with horseradish peroxidase-conjugated secondary antibody for 1 h at room temperature. Membrane blots were then developed with the ECL chemiluminescence system. Immunoreactive bands were captured on autoradiographic films and the densitometry of the bands was analyzed with Alpha Ease Image Analysis Software (Version 3.1.2).

### Immunoprecipitation

Proteins (500 μg in 500 μl) were pre-incubated with the protein G magnetic beads for 2 h. After gently centrifuging, the samples were incubated with 5 μg mouse GR antibody, ubiquitin antibody, or mouse IgG (as a negative control) for 12 h at 4 °C. After the addition of protein G magnetic beads for additional 1 h incubation, the beads were gently centrifuged and collected. After washing three times with lysis buffer, the beads were eluted by sample buffer containing Sodium Dodecyl Sulfate. Samples were collected and heated at 96 °C for 10 min for further western blot analysis.

### Immunostaining

Mice were perfused with 4% paraformaldehyde through the heart and brains were collected. After post-fixture and dehydration in 15% and 30% sucrose, brain tissues were embedded and cut into sections with a frozen cryostat. After washing with PBS (pH 7.4), brain sections were incubated with 0.03% hydrogen peroxide/methanol for 30 min to remove endogenous peroxidases and then permeabilized with 0.3% Triton X-100/3% bovine serum albumin/PBS for 60 min at room temperature. Sections were incubated with primary antibodies at 4 °C overnight. On the second day, brain sections were incubated with secondary antibodies and DAPI at 4 °C for 2 h. The following procedures were same as brain sections. All images were taken by using a Zeiss AxioImager-Z1 microscope with an AxioCamMR camera (Carl Zeiss, Oberkochen, Germany).

### Mouse corticosterone ELISA

For serum corticosterone determination, blood was collected via angulus oculi vessels of mice in heparinized tubes at 9–10 a.m. Corticosterone level was measured according to the instructions of an ELISA kit.

### Quantitative polymerase chain reaction (Q-PCR)

Total RNA was extracted from tissues and cells using RNeasy Plus Mini kit according to the instructions. Reverse transcription of cDNA was performed using the Transcriptor First Strand cDNA Synthesis Kit. Real-time PCR was performed using 10 μl of 2XSYBR Green PCR Master Mix, 1 μl of 20pM of specific primers, 1 μl cDNA and water to a final volume of 20 μl by using the 7500 Real-Time PCR system (Applied Biosystems, Foster City, CA, USA). GAPDH or actin was used as an endogenous control. Primer sequences were listed in Table S1 in the Supporting Information.

### Statistical analysis

All data were expressed as the mean ± SEM. Sample size and animal numbers were determined based on previous studies. Investigators were blind to group allocations for all experiments. Before the analysis, all data was checked for normality and homogeneity of variances. Differences between two groups were determined by Student’s *t*-test. The differences among groups were compared with one-way analysis of variance (ANOVA) followed by Tukey’s multiple-comparison tests or two-way analysis of variance (ANOVA) with repeated measures followed when appropriate by post hot tests using the Graphpad Prism 5.0 software. The variance is similar between the groups that are statistically compared. A value of *p* < 0.05 was considered statistically significant.

## Results

### Stress and glucocorticoid treatment reduced Ahi1 protein level

**Fig. 1:**
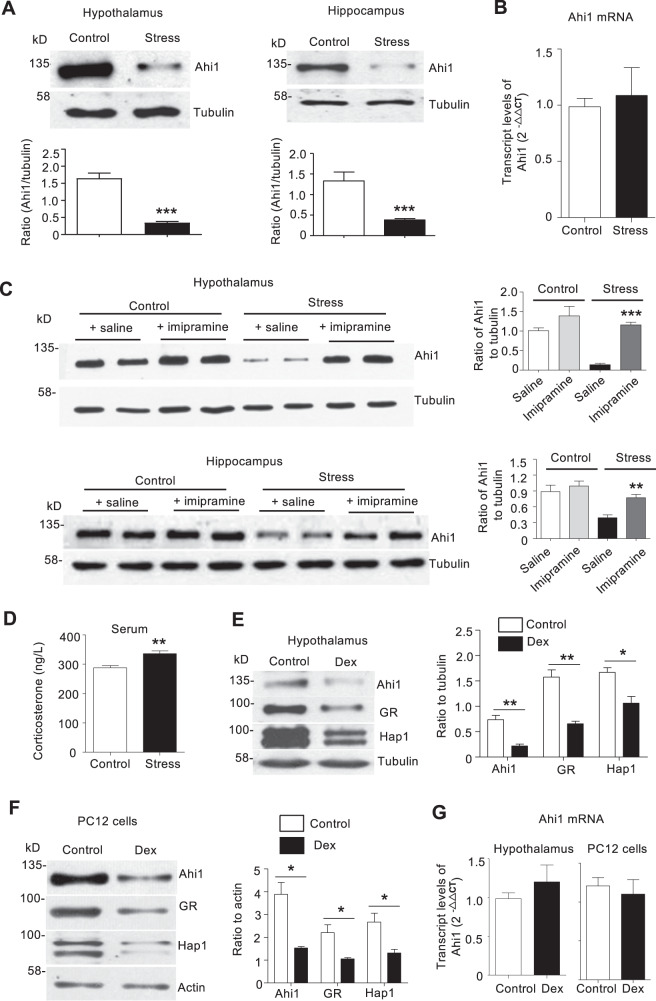
Stress and glucocorticoid treatment decreased Ahi1 protein level. **A** Western blotting analysis of Ahi1 expression in the hypothalamus and hippocampus of mice after spatial restraint stress for 30 days. The ratios of Ahi1 to β-tubulin were presented beneath the blots (hypothalamus: *t*_(8)_ = 8.208, ***P < 0.0001; hippocampus: *t*_(8)_ = 4.336, ***P = 0.0025). *n* = 5 mouse brains per group. **B** Ahi1 mRNA in the hippocampus was examined by quantitative PCR after spatial restraint stress for 30 days (*n* = 3 mouse brains per group). **C** Male ICR mice were treated without (Control) or with spatial restraint stress (Stress) for 4 weeks. After one week of stress, mice received antidepressant imipramine (i.p.) or saline (i.p.) daily for 3 weeks. After 3 weeks of treatment, the hypothalamus and hippocampus tissues were collected to analyze Ahi1 protein expression. β -tubulin was used as a loading control (*F*_(1,20) _= 5.829, ***P < 0.0001; *n* = 6 mouse brains per group). **D** Serum corticosterone level was assayed by an ELISA method after spatial restraint stress for 30 days. (*t*_(18)_ = 3.996, **P = 0.0008). *n* = 10 mice per group. **E** After Dex treatment for 21 days, Ahi1, Hap1, and GR levels in the hypothalamus was examined by western blotting (Ahi1: *t*_(4) _= 5.537, **P = 0.0052; Hap1: *t*_(4) _= 3.787, *P = 0.0193; GR: *t*_(4)_ = 6.031, **P = 0.0038) (*n* = 3 mouse brains per group). **F** PC12 cells were treated with Dex (20 μM) or normal saline (Control) for 72 h, Ahi1, Hap1, and GR in PC12 cell lysates were detected by western blotting (Ahi1: *t*_(4)_ = 4.428, *P = 0.0114; Hap1: *t*_(4)_ = 3.194, *P = 0.0331; GR: *t*_(4)_ = 3.308, *P = 0.0297. *n* = 3 cell samples). **G** Ahi1 mRNA levels were quantified by quantitative PCR in the hypothalamus samples (*n* = 3 mouse brains) from Dex-treated mice for 21 days (*t*_(4)_ = 0.9397, *P* > 0.05) or cell samples from Dex-treated PC12 (*n* = 3 cell samples) for 72 h, (*t*_(4)_ = 0.4929, *P* > 0.05). The transcript levels of Ahi1 were expressed as fold changes relative to GAPDH (mice) or actin (PC12 cells). All the data are presented as mean ± SEM.

Since conditional Ahi1 KO causes depressive phenotypes in mice^15,21^, it is unknown whether Ahi1 is reduced in brains of depressive mice. We used a spatial restraint stress mouse model and CSDS to examine Ahi1 expression. In the stressed mice, immobility times in TST and FST were significantly increased, and the consummation of 1% sucrose was markedly reduced after spatial restraint stress for 30 days, demonstrating the depressive behaviors (Supplemental Fig. 1A). Since Ahi1 is very abundant in the hypothalamus and is also expressed in the hippocampus^26^, Ahi1 expression was examined in these brain regions in the stressed mice. Western blotting revealed that Ahi1 expression was significantly decreased in the hypothalamus and hippocampus of stressed mice and CSDS mice (Fig. 1A and Supplemental Fig. 2). However, examining Ahi1 mRNA level via RT-PCR showed no significant change by spatial restraint stress, suggesting that stress-induced Ahi1 reduction occurs at the protein level (Fig. 1B). After spatial restraint stress for 7 days, the stressed mice were treated with imipramine (IM), a clinically used antidepressant, or saline for another 3 weeks. IM treatment significantly reversed stress-induced decrease of body weight, (Supplemental Fig. 1B), improved the immobility time in the TST and FST (Supplemental Fig. 1C), and restored sugar preference in stressed mice (Supplemental Fig. 1D). More importantly, IM treatment reversed the reduction of Ahi1 protein in the hypothalamus and hippocampus (Fig. 1C).

Previous studies suggest that serum glucocorticoid level is elevated in patients with MDD^27–29^. Similarly, glucocorticoid concentration was significantly increased in mice with spatial restraint stress (Fig. 1D). To determine whether the Ahi1 reduction is related to high-serum glucocorticoid in stressed mice, we used a synthetic glucocorticoid dexamethasone (Dex) to induce depressive phenotypes in mice, a well-established method of generating stressed animals^22,30,31^. As expected, Dex treatment for 21 days increased the immobility time in the TST and FST and decreased the consumption of 1% sucrose in the sucrose preference test (Supplemental Fig. 3A). We recently found that Huntingtin-associated protein 1 (Hap1), an Ahi1-interacting protein that stabilizes Ahi1^18,19,32^, could diminish the effect of Dex on the GR level in the hypothalamus^33^. We therefore examined the expression of Ahi1, Hap1, and GR in Dex-treated mice and found that all of them were reduced in the hypothalamus (Fig. 1E) and hippocampus (Supplemental Fig. 3B). To examine whether Dex directly regulates the expression of Ahi1, Hap1, and GR, we treated PC12 cells with Dex for 72 h and found that Ahi1, Hap1, and GR proteins were significantly decreased in parallel (Fig. 1F). However, Dex treatment did not alter the Ahi1 mRNA level in the mouse hypothalamus and PC12 cells (Fig. 1G).

### Dex and stress decreased the binding of GR to Ahi1 and reduced Ahi1

**Fig. 2:**
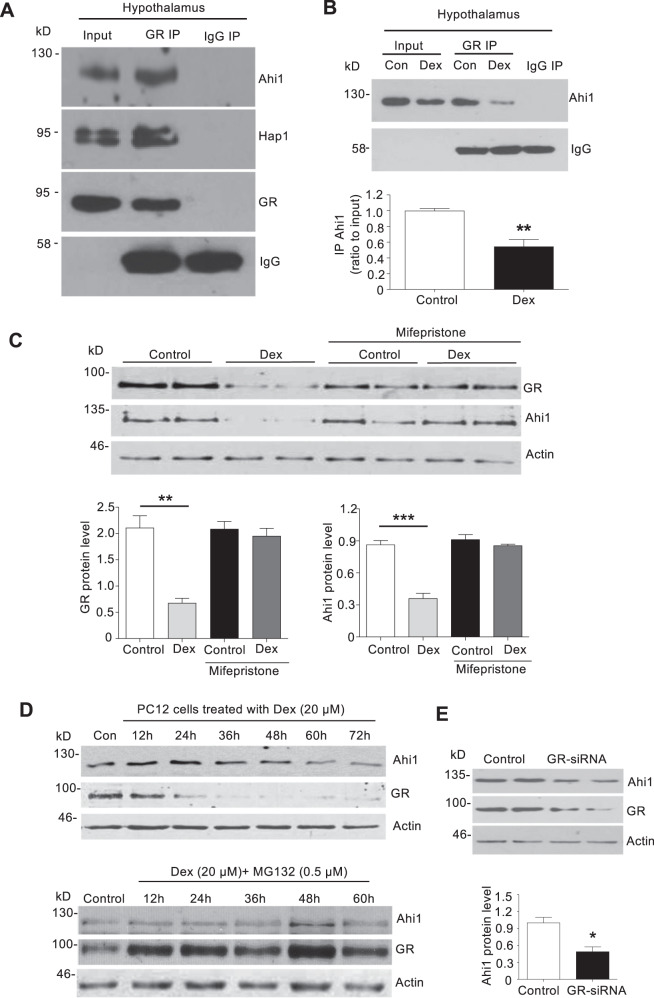
Dex decreased the binding of GR to Ahi1 and reduced Ahi1. **A** GR antibody and control IgG antibody were used for immunoprecipitation of the mouse hypothalamic tissue. GR-interacting proteins were examined by Western blotting with anti-Ahi1, anti-Hap1, and anti-GR. The representative images were shown. **B** After Dex treatment for 21 days, the hypothalamic tissues were collected for the immunoprecipitation by anti-GR or IgG. Ahi1 was examined by western blotting. The binding of GR to Ahi1 under Dex treatment was analyzed by using the ratio of immunoprecipitated Ahi1 to IgG. *N* = 4 mouse brains per group. *P* = 0.0028 versus Control (Con). **C** PC12 cells were treated with Dex (20 μM) with or without mifepristone (20 μM) for 72 h and then Ahi1 and GR were examined by western blotting. Quantitative analysis of Ahi1 and GR levels was further performed (Ahi1: *F*_(1,12)_ = 30.07, ***P < 0.0001; GR: *F*_(1,12)_ =16.99, **P = 0.0014). *n* = 4 cell samples. **D** PC12 cells were treated with Dex (20 μM) and MG132 (0.5 μM) for different times (0–60 h). Ahi1 and GR were examined by western blotting (*n* = 3 per group). **E** GR knockdown by GR-siRNA was performed in PC12 cells. Ahi1 and GR were examined by western blotting at 48 h after transfection and quantified. (*t*_(6)_ = 3.969, *P = 0.0166, *n* = 4 cell samples).

To test our hypothesis that Ahi1 and GR may form a protein complex in the cytoplasm to stabilize each other, we examined the interaction of Ahi1 and GR by performing immunoprecipitation. The results showed that Ahi1 was co-precipitated with GR and Hap1 from mouse hypothalamic tissues (Fig. 2A). Dex treatment (1 mg/kg for 21 days) (Fig. 2B) or chronic spatial restraint stress for 30 days (Supplemental Fig. 4) reduced the level of Ahi1 that was co-precipitated with GR in the mouse hypothalamus, consistent with the effect of Dex to reduce the level of Ahi1 (Fig. 1F). As Dex treatment induces the nuclear translocation of GR and reduces the cytoplasmic level of GR, we treated PC12 cells with mifepristone, a GR antagonist that can block GR nuclear translocations^1–3,34,35^. This treatment significantly reversed the Dex-induced reduction in GR and Ahi1 (Fig. 2C), suggesting that Ahi1 reduction is dependent on GR nuclear translocation.

It is known that Dex treatment can also induce GR degradation, which can be blocked by the proteasome inhibitor MG132^36–38^. We therefore treated PC12 cells with Dex at 20 μM, a concentration within the physiological range^39,40^, for different times in the presence of MG132. MG132 (0.5 μΜ) significantly blocked the degradation of GR and Ahi1 (Fig. 2D). To add direct evidence for the effect of GR to stabilize Ahi1, we reduced GR in PC12 cells via its small-interfering RNA (siRNA) and found that suppressing GR could also reduce Ahi1 (Fig. 2E). Since Ahi1 is a cytoplasmic protein and since cytoplasmic GR can be reduced by stress- or Dex-mediated nuclear translocation, the above results suggest that cytoplasmic GR level is critical for stabilizing Ahi1.

### Dex and stress promoted Ahi1 protein degradation through the ubiquitination of C-terminal Ahi1

**Fig. 3:**
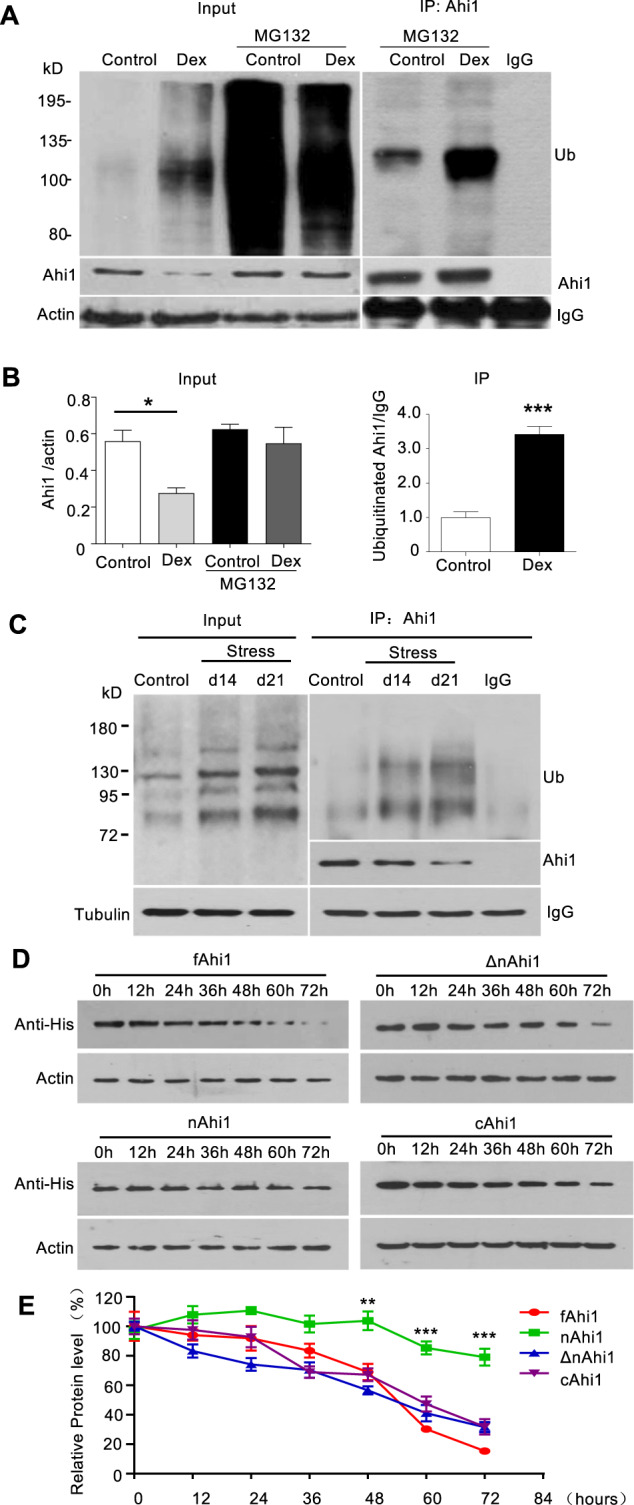
Glucocorticoid treatment and stress promoted Ahi1 protein degradation through ubiquitination. **A** PC12 cells were treated with or without Dex (20 μM) for 72 h, and then treated with or without MG132 (5 μM) for 10 h. Cell lysates were collected for the immunoprecipitation with Ahi1 antibody. Ahi1 and ubiquitinated proteins were detected with anti-Ahi1 and anti-ubiquitin antibodies. **B** The relative levels of Ahi1 (Ahi1/β-actin) (*F*_(1,8)_ = 3.341, *P = 0.0175) and ubiquitinated Ahi1 (Ub/IgG) (*t*(4) = 8.521, ***P = 0.001) were obtained from three independent experiments. **C** After spatial restraint stress for 14 days or 21 days, mouse hypothalamic tissues were collected for the immunoprecipitation with Ahi1 antibody. Ahi1 and ubiquitinated Ahi1 protein were examined by western blotting. **D** Different Ahi1-His fusion proteins expressing full length (fAhi1, aa 1–1047), N-terminal (nAhi1, aa 1–284), Ahi1 without N-terminal region (ΔnAhi1, aa 285–1047), and C-terminal (cAhi1, aa 651–1047) were transfected into PC12 cells. The co-transfected cells were treated with Dex (20 μM) for different times (0–72 h). Ahi1-His levels were determined by Western blotting with His antibody. **E** The degradation of different Ahi1-His fusion proteins (*F*_(3,84)_ = 55.50, ***P < 0.0001 versus control fAhi1, *n* = 4 cell samples).

To explore whether Ahi1 protein reduction is due to ubiquitination and degradation by the ubiquitin-proteasome system (UPS), we used Ahi1 antibody to perform immunoprecipitation of PC12 cells treated with Dex and MG132. The immunoprecipitation data showed that Dex treatment significantly increased the ubiquitination of the precipitated Ahi1 (Fig. 3A, B). Consistent with these results, ubiquitinated Ahi1 was also increased in the hypothalamus of stressed mice at 14 days and 21 days after spatial restraint stress (Fig. 3C). Therefore, these data suggested that stress or Dex-mediated GR nuclear translocation triggers the dissociation of GR from Ahi1 and the subsequent ubiquitination and degradation of Ahi1 by the proteasome enzymes.

To examine which region in Ahi1 is responsible for degradation by the UPS, we expressed different Ahi1 fragments fused to His in PC12 cells (Supplemental Fig. 5). These included full-length (fAhi1, aa 1–1047), N-terminal (nAhi1, aa 1–284), Ahi1 without N-terminal region (ΔnAhi1, aa 285–1047), and C-terminal (cAhi1, aa 651–1047). The transfected PC12 cells were treated with Dex (20 μM) for different times (0–72 h). Protein levels of different Ahi1 fragments were detected and quantified via Western blotting (Fig. 3C, D). The results indicate that N-terminal Ahi1 was resistant to degradation but all other Ahi1 fragments consisting of C-terminal domain were prone to rapid degradation, suggesting that C-terminal Ahi1 may facilitate the degradation of Ahi1 (Fig. 3E). Consistently, Dex treatment significantly increased the ubiquitination level of C-terminal Ahi1 (cAhi1) whereas the ubiquitination of N-terminal Ahi1 (nAhi1) remained unchanged (Supplemental Fig. 6).

### Ahi1 inhibits GR nuclear translocation and stabilizes GR in the cytoplasm

**Fig. 4:**
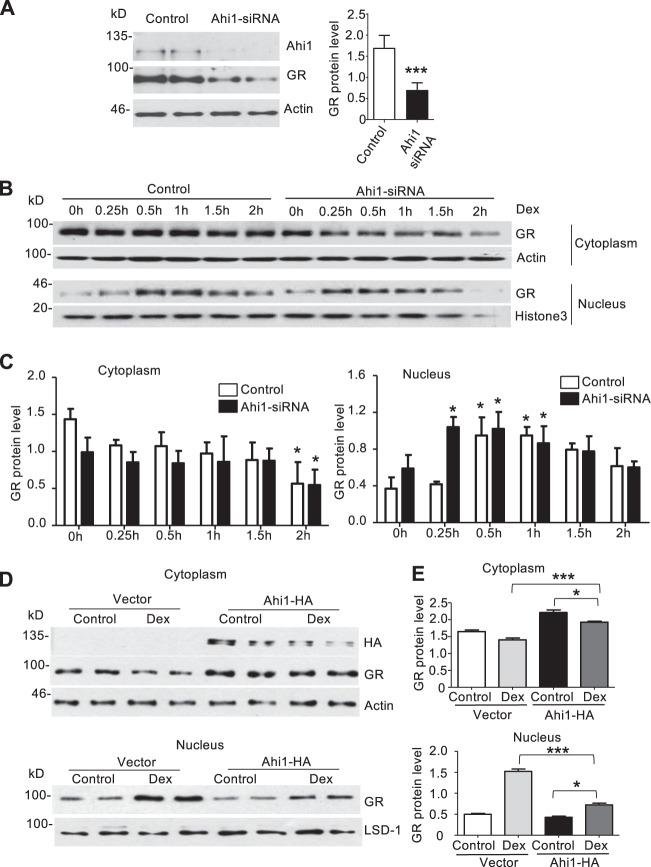
Ahi1 inhibits GR nuclear translocation and stabilizes GR in the cytoplasm. **A** Ahi1 knockdown was performed in PC12 cells by using Ahi1 siRNA. Ahi1 and GR levels were detected by western blotting at 48 h after siRNA transfection. (*t*(6) = 5.996, ***P = 0.001). *N* = 4 cell samples. **B** After Ahi1-siRNA transfection for 48 h, PC12 cells were treated with Dex (20 μM) for different time (0–2 h). Nuclear and cytoplasmic GR levels were examined by Western blotting. Histone-3 and actin were served as nuclear and cytoplasmic protein controls, respectively. **C** The ratios of GR protein to actin or histone-3. *P < 0.05 versus control group (*n* = 3 cell samples). **D** PC12 cells were transfected with PRK-Ahi1-HA plasmids or PRK vector for 48 h and then treated with Dex (20 μM) for 0.5 h. Cytoplasmic and nucleic fractions were isolated. GR was determined by western blotting in both fractions. LSD-1 and β-actin were used as loading controls of nuclear and cytoplasmic proteins. **E** The ratios (mean ± SEM) of GR to LSD-1 or actin (*n* = 4 cell samples). *P < 0.05, ***P < 0.001.

Given that GR can stabilize Ahi1, we wanted to examine whether Ahi1 also regulates GR level. To do so, we first knocked down Ahi1 in PC12 cells and verified that cytoplasmic GR was also significantly decreased in PC12 cells (Fig. 4A). Importantly, inhibition of Ahi1 promoted the nuclear translocation of GR in PC12 cells after Dex treatment, as GR was rapidly translocated into the nucleus at 15 min in Ahi1-siRNA-treated cells as compared with increased GR nuclear translocation at 30 min in control cells (Fig. 4B–C). Furthermore, Ahi1 over-expression markedly inhibited Dex-induced GR nuclear translocation at different time points (Fig. 4D). To verify this result, we analyzed GR distribution in the cytoplasm and the nucleus at 30 min after Dex treatment. Ahi1 over-expression reduced nuclear GR level, but significantly increased GR level in the cytoplasm (Fig. 4E). These results clearly demonstrated that Ahi1 plays a critical role in modulating GR nuclear translocation and maintaining its stability in the cytoplasm.

### Ahi1 deficiency increased nuclear GR in the brain and the hyposensitivity to antidepressant treatment

**Fig. 5:**
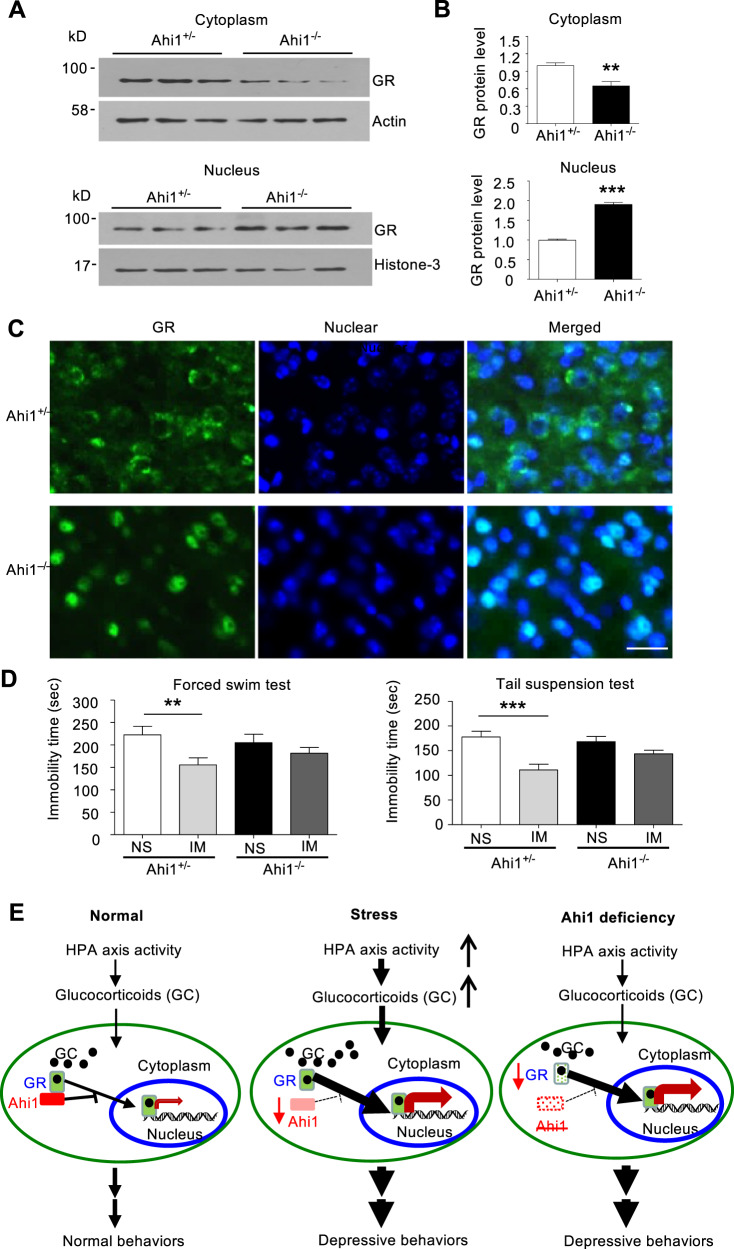
Ahi1 deficiency increased nuclear GR level and reduced the response to antidepressant treatment in Ahi1 KO mice. **A** Cytoplasmic and nuclear GR in the hypothalamus of Ahi1^−/−^ and Ahi1^+/−^ mice was examined by Western blotting. Cytoplasmic protein β-actin and nuclear protein histon-3 served as controls. **B** The protein level of GR was expressed as fold changes relative to β-actin (**P = 0.002) or histone-3 (***P < 0.0001) versus control (Ahi1^+/−^ mice. **C** Immunofluorescent staining of the hypothalamic sections from control (Ahi1^+/−^) mice and Ahi1 KO (Ahi1^−/−^) mice. GR (green) and the nuclei (blue) were seen in the merged images. Scale bar: 20 μm. **D** Homozygous (−/−) and heterozygous (+/−) Ahi1 KO mice received spatial restraint stress treatment 2 h daily for 4 weeks. From the second week, mice were treated (i.p.) with imipramine (IM) or normal saline (NS) daily for 3 weeks. Behavioral tests TST and FST were performed. (TST: *F*(1,55) = 4.070, ***P < 0.0001 versus saline-treated Ahi1^+/−^+NS mice group; FST: *F*(1,55) = 1.707, **P = 0.0093 versus saline-treated Ahi1^+/−^+NS mice group). (*n* = 14–15 mice per group). **E** The proposed diagram of the modulation of Ahi1 protein on stress-mediated GR signaling. In normal conditions, glucocorticoid (GC) binds to its receptor GR and enters the nucleus. Ahi1 binds GR to prevent its nuclear translocation (left). Under chronic stressful conditions, high HAP axis activity releases glucocorticoid (GC) that binds to GR to lead to its nuclear translocation and regulation of depression-related gene expression. The nuclear translocation of GR reduces Ahi1 stability (middle). When Ahi1 is absent or deficient, more GR is translocated to the nucleus, and this increased basal stress response reduces response to antidepressants (right).

To provide in vivo evidence for the role of Ahi1 in regulating GR expression, we examined GR expression in the hypothalamic tissues of Ahi1 KO mice. Western blotting revealed that cytoplasmic GR level was significantly decreased in homozygous Ahi1 KO (Ahi1^−/−^) mice compared with control heterozygous (Ahi1^+/−^) mice (Fig. 5A). In contrast, nuclear GR was significantly increased in the hypothalamus of Ahi1 KO mice (Fig. 5A). These differences were verified by quantifying the ratios of GR to the cytoplasmic and nuclear marker proteins (Fig. 5B). Immunofluorescence staining further demonstrated that Ahi1 KO (Ahi1^−/−^) mice had the lower GR level in the cytoplasm and the higher GR level in the nucleus than control mice (Fig. 5C). Since the therapeutic effects of antidepressants are dependent on the nuclear translocation of GR and nuclear GR-mediated gene transcription, the abnormally subcellular distribution of GR in Ahi1 KO mouse brain may alter animal’s response to antidepressants. We therefore used imipramine to treat Ahi1 KO and control mice after spatial restraint stress for 1 week. After 3 weeks of stress and treatment, imipramine significantly decreased the immobility time in control mice, but not in Ahi1 KO mice (Fig. 5D). Based on our findings, we proposed that GR and Ahi1 stabilize each other in the cytoplasm. Extensive GR nuclear translocation by glucocorticoid-like ligand treatment or stress reduces the binding of GR to cytoplasmic Ahi1 and promotes the nuclear translocation of GR, resulting in the ubiquitination and degradation of Ahi1. Conversely, Ahi1 reduction decreases the cytoplasmic GR level and promotes ligand-dependent GR nuclear translocation, thereby modulating stress-induced depressive phenotypes and, potentially, the therapeutic effects of antidepressants (Fig. 5E).

## Discussion

The HPA axis is a critical endocrine system for dealing with acute and chronic stresses from the environment, and dysfunction of the HPA axis is well documented in many psychiatric disorders, including MDD^41^. Stress causes the release of glucocorticoid hormones from the adrenal cortex, which binds GR to mediate the nuclear translocation of GR to trigger stress response signaling pathways^1–3^. Acute or chronic stress, which could increase glucocorticoid hormones, can reduce GR levels in the peripheral tissues^42–44^ and postmortem brains^45^ of patients with depression. Consistently, glucocorticoid administration in rodents increases the cortisol concentration in blood to reduce GR level and/or function, resulting in depressive-like behaviors^4,31,33,46,47^. Increasing evidence suggests that GR may be associated with neurotransmitter synthesis, which may be the reason for GR’s ability to regulate depression^48,49^.

Our study provides the following findings to support a role for Ahi1 in stress-mediated-nuclear translocation of glucocorticoid receptor. First, the level of Ahi1 is reduced in the brain tissues of stressed mice. Second, Ahi1 interacts with GR to regulate its nuclear translocation. As Ahi1 and Hap1 form a stable protein complex^18^, the interaction of Ahi1 and GR is consistent with our recent finding that Hap1 also plays a role in stabilizing cytoplasmic GR^33^. Third, Ahi1 deficiency causes abnormally increased nuclear distribution of GR in mice and attenuates their responses to antidepressants.

The above findings were all based on the evidence for the interaction of Ahi1 with GR, which stabilizes GR in the cytoplasm. In addition, overexpressed Ahi1 stabilizes GR in the cytoplasm to prevent GR from entering the nucleus. Furthermore, stress-induced reduction of GR in the cytoplasm destabilizes cytoplasmic Ahi1, thereby decreasing Ahi1 levels that are reversible by antidepressant treatment. Additional evidence also includes the treatment with mifepristone, a GR antagonist, which could prevent the degradation of Ahi1 after Dex treatment.

An estimated 40% of patients are unable to respond to the first line of antidepressant treatment^47,50^. Growing evidences show that the therapeutic response to antidepressants can be achieved by the restoration of disrupted GR function in patients with MDD^6^. GR mutations and modifications (such as phosphorylation), GR-interacting proteins (such as FKBP5), and GR antagonists (such as mifepristone) have received great attention for their regulation on MDD^1,35,51–53^. For example, FKBP5, an immunophilin protein, inhibits GR nuclear translocation by competing with GR’s binding to cortisol^54^, and its polymorphisms are associated with major depressive disorders^55–58^. Similarly, genetic studies also show an association between Ahi1 variants and depression^14^. Consistently, our early study showed that homozygous Ahi1 KO mice display depressive behavior^15^. However, heterozygous Ahi1 knockout (Ahi1^+/−^) mice revealed an attenuated anxiety response on various relevant paradigms and were found to be hyposensitive to stress^17,59,60^. In the present study, we found that the cytoplasmic level of Ahi1 is important for modulating nuclear GR level and subsequent responses to stress. Since Ahi1 binds cytoplasmic GR to stabilize each other and since Ahi1 deficiency can reduce cytoplasmic GR level, Ahi1 deficiency would diminish the effects of antidepressants whose action relies on the level and activation of GR^6,7^. Our findings revealed the association of decreased cytoplasmic GR levels with a resistance to the antidepressant IM in Ahi1 KO mice, providing the potential mechanism of antidepressant-resilient depression. Similar to other findings of a different Ahi1^+/−^ mouse line that was generated via gene trap technology^16,17^, western blotting did not reveal significant difference in Ahi1’s expression in our adult Ahi1^+/+^ and Ahi1^+/−^ mouse brains. However, unlike our Ahi1^+/−^mice that were generated via the Cre-Loxp system and showed no anxious behaviors^15^, attenuated anxiety response on various relevant paradigms was reported in Ahi1^+/−^ mice generated by gene trap targeting^17,59,60^. Thus, phenotype differences between two types of Ahi1^+/−^ mice are likely due to different genetic backgrounds and/or genetic targeting approaches. In summary, our studies suggest that coordinated interaction of Ahi1 with GR regulates the normal behaviors and that the imbalance of Ahi1 expression results in depressive phenotypes by impaired GR signaling. Therefore, Ahi1 could be a therapeutic target for the treatment of stress-mediated depressive disorders.

## Supplementary information

Supplemental information
