# Supplementary material for: Ahi1 regulates the nuclear translocation of glucocorticoid receptor to modulate stress response
Source: Transl Psychiatry. 2021 Mar 29;11:188. doi: 10.1038/s41398-021-01305-x (PMC8007735; doi:10.1038/s41398-021-01305-x)
Supplement: Supplementary file 1 — Supplemental information [file 41398_2021_1305_MOESM1_ESM.pdf]

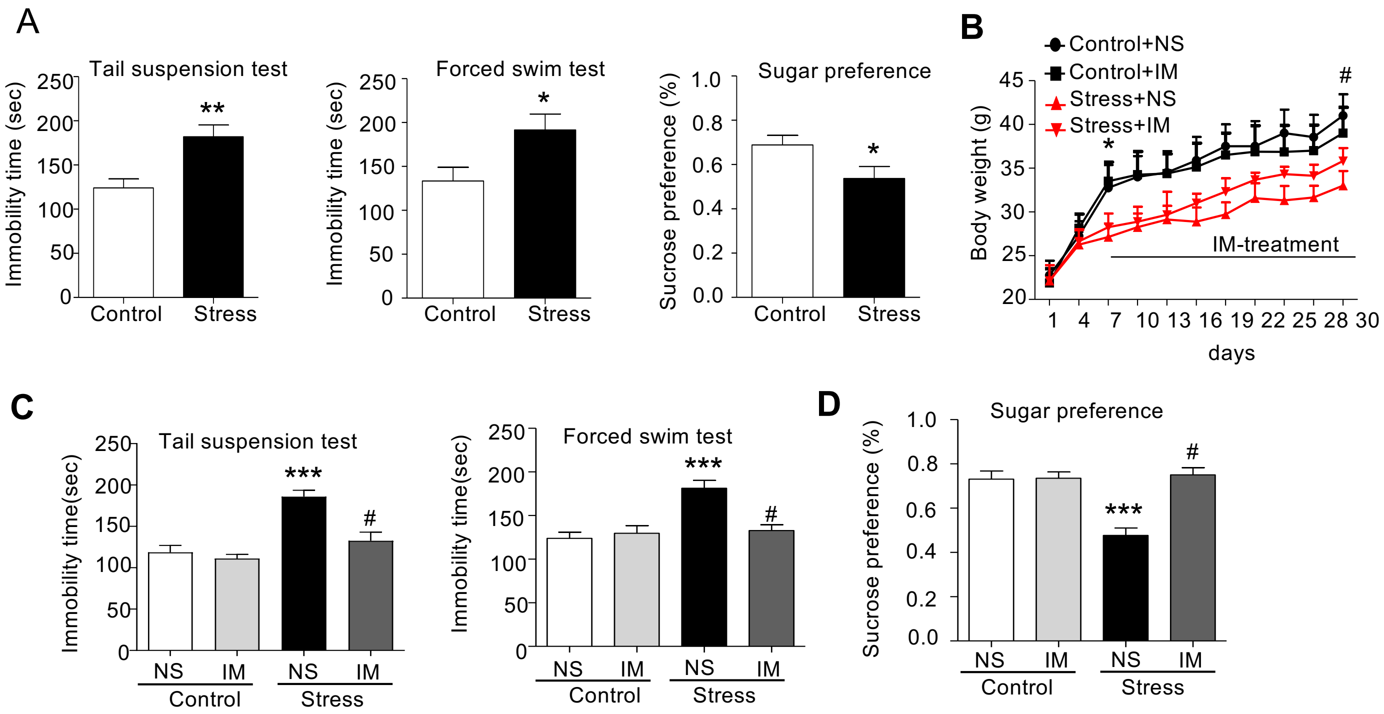

Figure S1. Spatial restraint stress induced depressive-like behaviors and that was reversed by antidepressant imipramine

A. Male ICR mice were given spatial restraint stress (Stress) for 30 d. Behavioral tests (TST, FST, and SPT) were performed (TST: $t_{(18)}$=3.452, ***P*=0.0028 versus control; FST: $t_{(18)}$=2.414, **P*=0.0266 versus control; SPT: $t_{(16)}$=2.178, **P*=0.0429 versus control; n=10 mice per group).

B. Male mice received spatial restraint stress for 2 h daily for 4 w. From the second week, mice were treated (i.p.) with imipramine (IM, 20 mg/kg body weight) or normal saline (NS) daily for 3 weeks. Body weight was recorded . n=8-10 mice per group. **P*<0.05, Control+NS group versus Stress+NS group; $^{\#}P$<0.05, Stress+IM group vs Stress+NS group.

C. After treatment, behavioral tests including TST and FST (TST: $F_{(1,80)}$ =7.121, ****P*<0.0001; FST:$F_{(1,80)}$ =11.42, ****P*<0.0001 versus Control+NS group; $^{\#}P$<0.05 versus Stress+NS group). n=18-24 mice per group.

D. Sugar preference test (n=8-10 mice per group) was performed ($F_{(1,32)}$=16.24, ****P*=0.0003 versus Control+NS group. n=18-24 mice per group; $^{\#}P$<0.05 versus Stress+NS group).

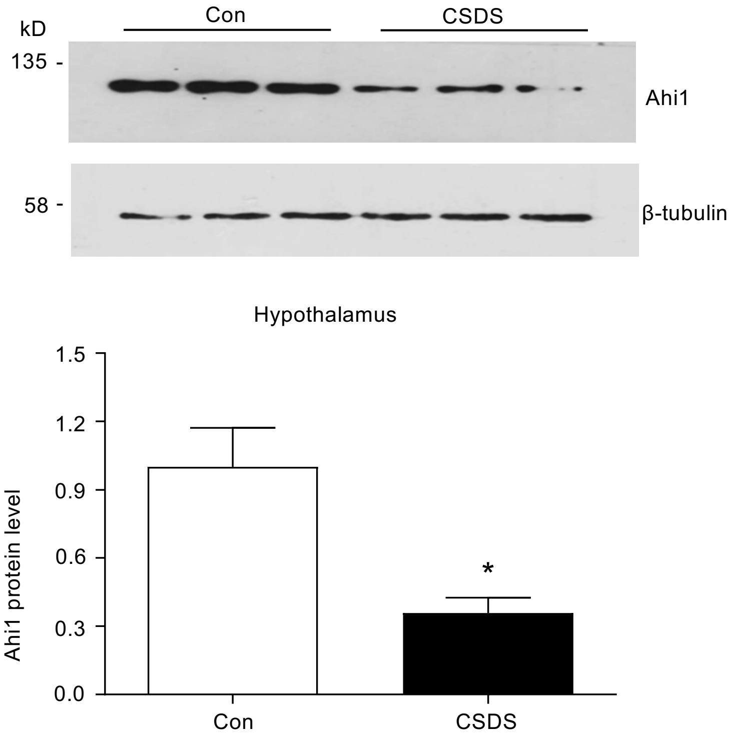

Figure S2. Chronic social defeat stress (CSDS) caused the decrease of Ahi1 in the hypothalamus in mice.

A. After 10 days of social defeat stress, mice were scarified and the hypothalamus was collected.

B. Ahi1 protein in hypothalamus was detected by Western blotting and quantitative analysis was performed. β-tubulin was used as a loading control ($t(4)=3.479$, $p=0.0254$; $n=3$ mouse brains per group). *$p<0.05$ versus Control.

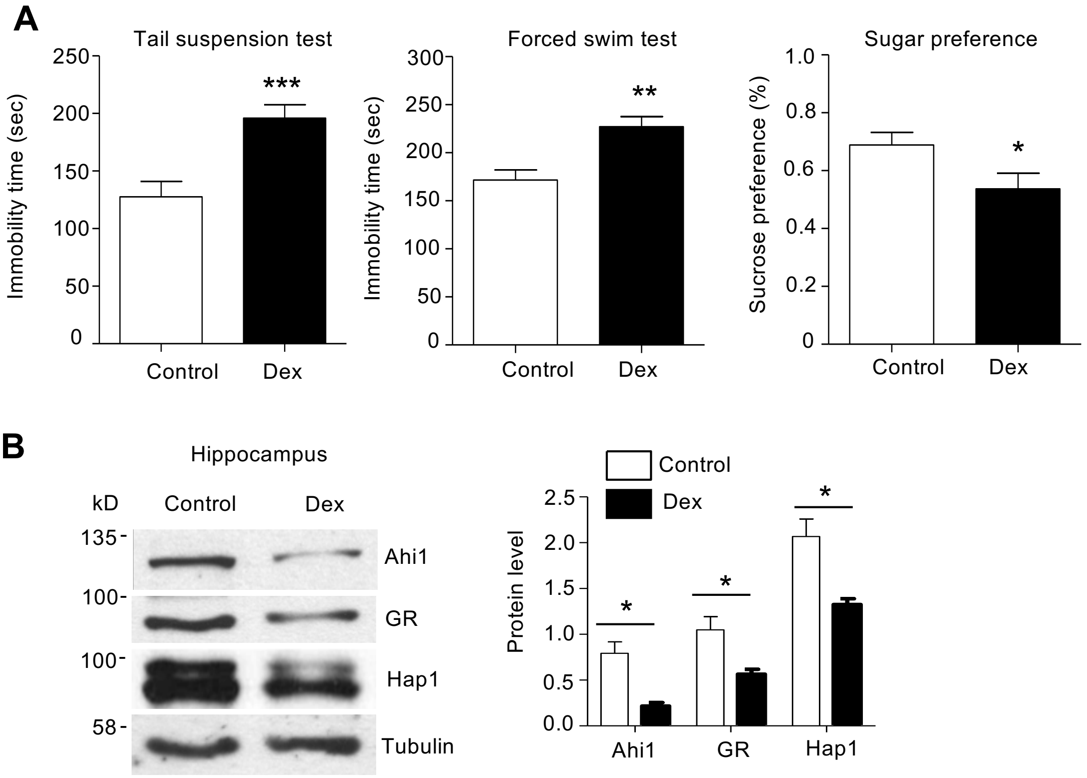

Figure 3. Ahi1 protein level decreased in glucocorticoid-treated mice with depressive behaviors

A. Male mice were treated with dexamethasone (Dex, 1mg/kg) for 21 d and behavioral tests (TST, FST, and SPT) were carried out（TST: $t_{(18)}$ =3.825, ****P*=0.0012; FST: $t_{(18)}$=3.665, ***P*=0.0018; SPT: $t_{(18)}$=2.178, **P*=0.0429, n=10 mice per group).

B. After Dex treatment for 21d, Ahi1, Hap1, and GR levels in the hippocampus was examined by Western blotting (Ahi1: $t_{(4)}$= 4.378, **P*=0.0119; Hap1: $t_{(4)}$=3.678, **P*=0.0212; GR: $t_{(4)}$=3.175, **P*=0.0337; n=3 mouse brains per group).

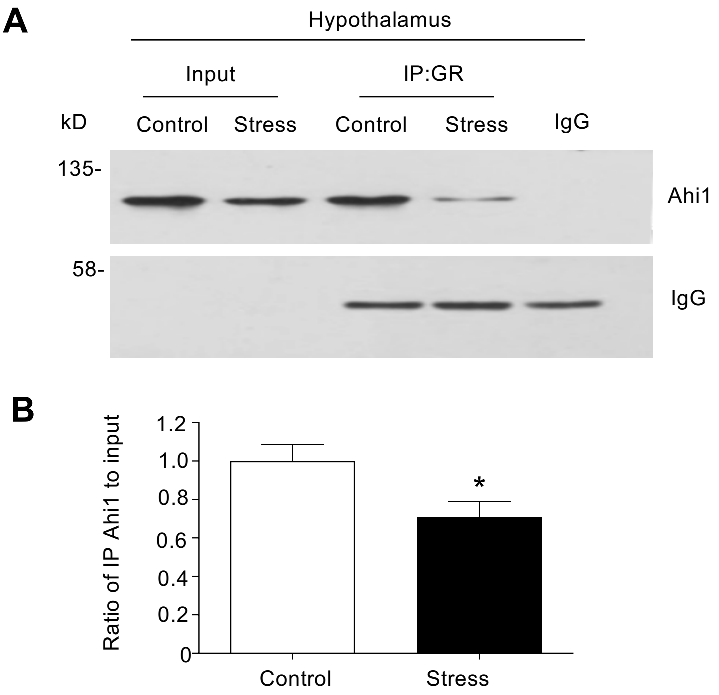

Figure S4. Stress reduced the association of Ahi1 with GR
A. After spatial restraint stress for 30 d, the mouse hypothalamic tissues were collected for the immunoprecipitation with GR and IgG antibodies. Ahi1 was examined by western blotting.
B. The binding of GR to Ahi1 under spatial restraint stress was analyzed by using the ratio of immunoprecipitated Ahi1 to IgG ($t_{(6)}$=2.482, *P=0.0477; n=4 mouse brains per group).

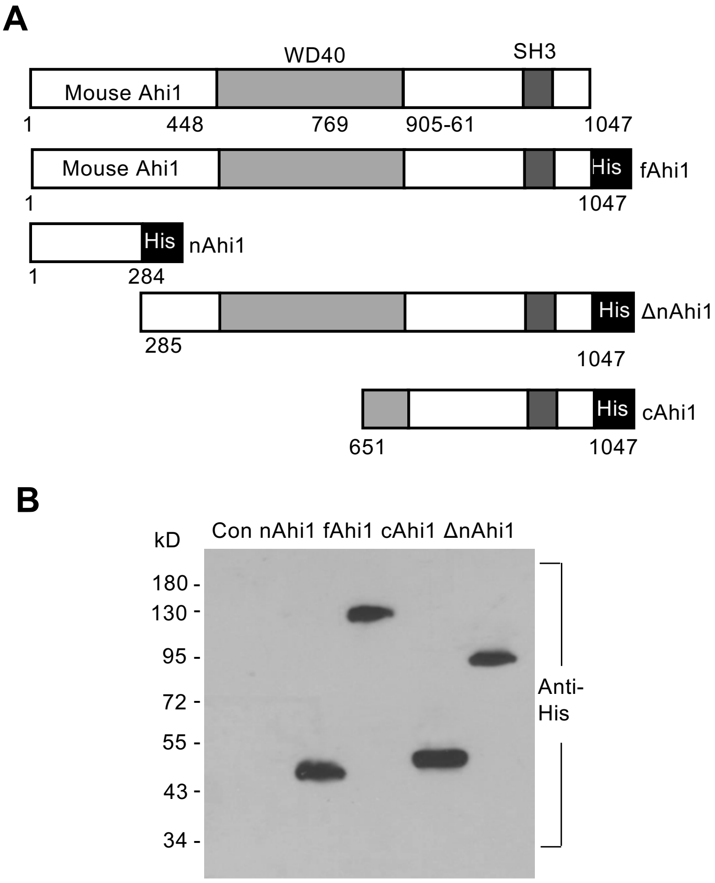

Figure S5. Structures of different Ahi1-His fusion proteins

A. Ahi1-His expressing full length (fAhi1, aa 1-1047), N-terminal (nAhi1, aa 1-284), Ahi1 without N-terminal region (ΔnAhi1, aa 285-1047), and C-terminal (cAhi1, aa 651-1047) Ahi1 proteins.

B. PC12 cells transfected with vector plasmid or different Ahi1-His fusion plasmids were detected by Western blotting with anti-His antibody.

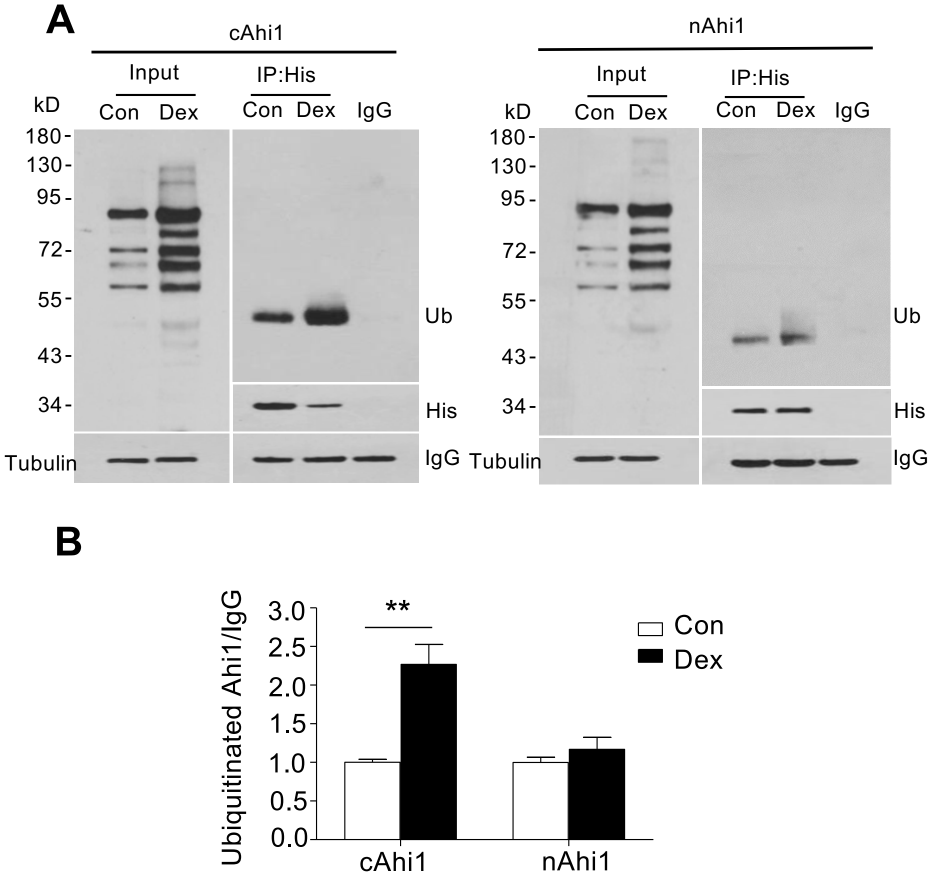

Figure S6. Ubiquitination of C-terminal Ahi1

A. PC12 cells were transfected with c-Ahi1 or nAhi1 plasmid for 48 h and treated with or without Dex for 72 h. Cell lysates were collected for the immunoprecipitation with anti-His antibody. Different Ahi1 fragments and ubiquitinated proteins were detected by anti-His and anti-ubiquitin antibodies.

B. The relative levels of ubiquitinated Ahi1 fragments (ratio to IgG) were obtained from three independent experiments (cAhi1: $t_{(4)}$=4.898, ***P*=0.0081; nAhi1: $t_{(4)}$=1.017, *P*=0.3666).

Supplementary Table 1
PCR primers used in the study

Gene name	Forward	Reverse
Ahi1(Mouse)	GACAGGAGAACAAGTGGCAATG	ATCAGTGGTCAGCACGAACGA
Ahi1(Rat)	GGGAGTCTACATTCACCGAACC	AGTAAGATGAGACGGGACGCTC
AHI1 isoforms I（Human）	GCTCCTCAAAAGCAATCAATCAA	CTCATTTCAGAATGTGTCATAGAT
AHI1 isoforms Ⅱ（Human）	CATGCTGACCGCTCAAGAGATT	GTGTTGAATTCAGCAAAGTGACT
AHI1 isoforms III（Human）	CCTGAGATAAAGGAGAGATCCC	GAAGGAGGTGTCTCTGTGAGCT
AHI1 isoforms I, II ,III（Human）	CTGTCACAGAGGTGATACGTTC	GACTGTTGTGAGGAAACTGCTG
Gapdh(Mouse)	CATGGCCTTCCGTGTTCCTA	CTTCACCACCTTCTTGATGTCATC
Actin(Rat)	CCTAAGGCCAACCGTGAAAAG	GCTCGAAGTCTAGGGCAACATAG
Gapdh forward （Human）	CTTCTCCATGGTGGTGAAGAC	CCCATCACCATCTTCCAGGAG
